# Reclaiming Agency in Care Decisions and Barriers From the Perspectives of Individuals With Acquired Brain Injury and Their Family Members

**DOI:** 10.1111/hex.14109

**Published:** 2024-06-14

**Authors:** Tamara Ownsworth, Annerley Bates, Kerrin Watter, Clare Morgan, Ryan Bell, Janelle Griffin, Ben Turner, Areti Kennedy, Melissa Kendall, Belinda Adams, Emily Gibson, Troy Hakala, Jessie Mitchell

**Affiliations:** ^1^ The Hopkins Centre, Menzies Health Institute Queensland Griffith University Brisbane Queensland Australia; ^2^ School of Applied Psychology Griffith University Brisbane Queensland Australia; ^3^ Division of Rehabilitation Metro South Health Hospital and Health Service Brisbane Queensland Australia

**Keywords:** acquired brain injury, agency, person‐centred, rehabilitation, self‐advocacy

## Abstract

**Background:**

The ability to self‐advocate or have a say in one's care is integral to personalised care after acquired brain injury (ABI). This study aimed to understand what constitutes self‐advocacy and associated barriers and facilitators throughout hospital transitions and into the community.

**Method:**

Qualitative methodology was employed with semistructured interviews conducted with 12 people with ABI and 13 family members. Interviews were conducted at predischarge (in‐person or via telephone) and 4 months postdischarge (via telephone) from the brain injury rehabilitation unit of a tertiary hospital. Data were thematically analysed using a hybrid deductive–inductive approach.

**Results:**

Self‐advocacy reflects the process of *reclaiming agency* or people's efforts to exert influence over care decisions after ABI. Agency varies along a continuum, often beginning with impaired processing of the self or environment (*loss of agency*) before individuals start to understand and question their care (*emerging agency*) and ultimately plan and direct their ongoing and future care (*striving for agency*). This process may vary across individuals and contexts. Barriers to self‐advocacy for individuals with ABI include neurocognitive deficits that limit capacity and desire for control over decisions, unfamiliar and highly structured environments and lack of family support. Facilitators include neurocognitive recovery, growing desire to self‐advocate and scaffolded support from family and clinicians.

**Conclusion:**

Self‐advocacy after ABI entails a process of reclaiming agency whereby individuals seek to understand, question and direct their ongoing care. This is facilitated by neurocognitive recovery, growing capacity and desire and scaffolded supports. Research evaluating approaches for embedding self‐advocacy skills early in brain injury rehabilitation is recommended.

**Patient or Public Contribution:**

Two caregivers with lived experience of supporting a family member with ABI were involved in the design and conduct of this study and contributed to and provided feedback on the manuscript.

## 1 Introduction

People who sustain an acquired brain injury (ABI), or brain damage caused by events after birth [1], will generally endure multiple care transitions involving different care levels and locations in hospital and between community services [2]. For individuals with ABI and their families, the trajectory has been described as the ‘ultimate endurance test’ [3] due to the need to navigate multiple complex systems and decision points to mobilise appropriate support [2, 4]. Compounding this challenge, the ability of individuals to self‐advocate and participate in care decisions is often compromised by postinjury cognitive communication and behavioural changes [5]. Yet, there is currently a lack of understanding of what constitutes effective self‐advocacy after ABI and associated barriers and facilitators.

Xie et al. [6] identified that individuals with acquired neurological disability experienced, on average, eight care transitions in the first 12 months postdischarge. Care transitions are known points of vulnerability for people with ABI due to their diverse and complex support needs [2]. Hospital discharge entails navigating a safe discharge and identifying an individualised mix of supports from funding sources [7, 8]. Lack of effective planning can contribute to discharge delays or adverse outcomes, including secondary complications and hospital readmissions [4]. Readying people with ABI and their families to be knowledgeable experts within the system can contribute to more personalised care transitions and better outcomes [2].

Supporting people with ABI to identify, communicate and enact their rights and choices across care transitions ultimately requires a multifaceted approach that enables self‐advocacy at personal and system levels. Person‐centred care principles emphasise the importance of transition planning occurring in a manner that promotes personal choice and control [5]. Yet, participation in these critical decision‐making processes is reliant upon individuals' capacity for self‐advocacy. Self‐advocacy requires knowledge of one's self (i.e., needs, injury impact, goals) and rights, and the ability to express and assert one's rights and choices within relevant decision‐making contexts [9].

Most adults with ABI had formerly established independence with decision‐making. However, they find themselves thrust into a situation requiring them to navigate complex systems in which they typically have no previous experience. Challenges of this adjustment process are compounded by the diverse impairments arising from ABI that can lead to profound changes in self‐advocacy skills [5]. For example, self‐awareness impairments affect individuals' capacity to recognise postinjury changes and understand their future implications [10]. Moreover, cognitive deficits may reduce people's ability to remember new information and make considered decisions and plans regarding their own needs [5]. Communication impairments affect comprehension skills and the ability to express one's preferences and choices [11].

Due to these combined factors, the emergence of self‐advocacy skills after ABI can take considerable time and may not occur until individuals have been living in the community for many years, if at all. Consequently, research has focused on the role of clinicians and family members as advocates and communication partners [12]. Person‐centred practice frameworks advocate the importance of ‘supporters’ and ‘supported decision‐making’ enabling individuals with ABI to be increasingly involved in goal‐setting and decision‐making [5]. Re‐engaging with choice and control in the community after ABI has been described as a dynamic and challenging process, requiring clinicians and supporters to facilitate a ‘gradual and negotiated return to agency’ [13, p. 2710].

One strategy used in rehabilitation to enhance sense of choice and control is person‐centred goal setting for community integration [14]. Contextual factors promoting individuals' participation in goal‐setting and decision‐making include supporters having a positive attitude towards self‐advocacy, creating decision‐making opportunities and adjusting support according to individuals' emerging capacity and abilities [5]. Early introduction of self‐management approaches has been advocated [15, 16], and largely focus on staff training and resources to promote individuals' understanding of ABI and self‐efficacy.

Self‐advocacy skill development is related to but extends beyond person‐centred and collaborative goal setting [14] and self‐management approaches [15, 16], with a broader focus on supporting self‐knowledge and understanding and asserting one's rights and choices within decision‐making contexts. It is currently unknown to what extent individuals with ABI feel able to self‐advocate, or to identify, express or enact their needs, during early care transitions as needed to participate in decision‐making. Accordingly, this study aimed to understand what constitutes self‐advocacy and identify associated barriers and facilitators during early care transitions.

## 2 Methods

### 2.1 Design

Informed by a phenomenological approach [17], semistructured interviews were conducted on two occasions to understand individuals' lived experiences in a specific care context. Individuals with ABI and their family members were interviewed predischarge (T1) and approximately 4 months postdischarge (T2) from the inpatient brain injury rehabilitation unit of a single tertiary hospital.

### 2.2 Participants

Following ethical clearance and informed consent procedures, 12 individuals with ABI (traumatic brain injury [TBI] and stroke) and 13 family members were recruited. Purposive sampling [17] was used to identify participants with diverse characteristics that may influence care needs and self‐advocacy (e.g., age, sex, traumatic vs. nontraumatic ABI, time since injury, metropolitan vs. rural residence). Eligibility criteria for individuals with ABI included a minimum 2‐week stay and expected discharge in 1–2 weeks, capacity to provide informed consent and adequate receptive and expressive communication with appropriate assistance. Eligibility criteria for family members included those aged ≥18 years and involved in caring for a participant with ABI.

### 2.3 Data Collection

T1 interviews focused on perceptions of the ability of the individual with ABI to identify and express their needs during hospital‐based care transitions (Table 1). T2 interviews sought reflections on to what extent and how individuals expressed or enacted their needs during and since discharge (Table 1). In this context, the care continuum involved inpatient, outpatient and community‐based specialist rehabilitation services [18]. Questions were collaboratively developed by the research team, including multidisciplinary ABI clinicians and two researchers with lived experience of caring for relatives with ABI. Participants received questions before each interview. Guidelines for conducting interviews with people with ABI were followed [19], including the use of closed questions before elaboration prompts. Participants with ABI were encouraged to have a communication partner (e.g., family member) to rephrase questions using familiar language.

**Table 1:** Interview topic and question guide for T1 predischarge interviews and T2 postdischarge interviews.

Interview topics	Main questions and example prompts
*T1 predischarge interviews*	
Type of care transitions experienced	Since your brain injury, which hospitals have you been in? (If two or more hospitals) What was it like moving from one to the other? – What positive experiences or memories do you have? – What negative experiences or memories do you have? Since your brain injury, can you tell us the different wards you have been in? It's ok if you don't remember them all.
Experience of care transitions	When you have moved between wards in the hospital, what was this like? – What positive experiences or memories do you have? – What negative experiences or memories do you have? Can you think of anything that could have made these changes in care better for you?
Experiences of being involved in decisions and having a say about changes in care	When you moved between wards or hospitals, did you feel you could tell people what you needed or wanted? – If yes—How did you do this? What helped you have your say? – If no—What stopped this from happening? What made this hard? – How well did you understand what was going to happen when you moved? – Did you have the chance to ask questions about what was going to happen? – How much chance did you feel you had to tell people what you needed or wanted when planning your change in care?
Current involvement in decisions and discharge and support planning	Are you involved in planning for your discharge and planning for the future? – If yes—How are you doing this? – If no—What is stopping this from happening? – Do you know what is happening with your plan for discharge and future support after discharge? – Who is involved in making plans for your discharge and future support? – Has there been anything that has held up your discharge or ability to go home? – How well have you been able to tell people what you need or want?
Overall perceptions of care and having a say in care	What things helped you or made it easier to have your say when moving hospitals or wards? What things did not help you or made it harder to have your say when moving hospitals or wards? How well do you feel that different people in hospital understand brain injury and the effects?
Importance of having a say in your care	Is having a say in your rehabilitation and care important to you? – If yes—Why is this important to you? What parts are important to you? – If no—Can you tell me why this is the case? What does having your say (in your care and changes to your care) mean to you? How would you know that you were being heard? Overall, how would you sum up your stay?
*T2 postdischarge interviews*	
Reflection on experience of hospital discharge	What was it like for you to leave hospital? – What positive experiences or memories do you have? – What negative experiences or memories do you have? – How involved were you in the plans for leaving hospital? – How much did you feel that you were able to have a say in your plans for discharge? – How well did you feel you were able to tell people what you needed or wanted about your future support and rehabilitation? – Looking back, is there anything that could have made leaving hospital easier or better for you?
Experiences of being involved in decisions about changes in care since leaving hospital	After you left hospital, did you feel you could tell people what you needed or wanted? – If yes—How did you do this? – If no—What stopped this from happening?
	Can you tell me about some of the changes in your care and rehabilitation since leaving hospital? What services and support have you received? Were you involved in making choices or decisions about these changes? (Think about just one of these changes if it is easier) – If yes—How were you involved? – If no—What stopped this from happening?
	Think about when you experienced changes in your care and rehabilitation after hospital. Did you feel you could tell people what you wanted or needed? – If yes—How did you do this? – If no—What stopped this from happening? How well do you feel that people in different services understand brain injury and the effects?
Your future care and rehabilitation	Is having a say in your rehabilitation and care important to you? – If yes—Why is this important? – If no—Why not? In thinking about your future rehabilitation and care, what is important to you? – Why is this important to you?
Advice for others	If you met someone who was 1–2 weeks away from being discharged from hospital, what advice, if any, would you offer?

*Note:* Family members' interviews referred to ‘your relative [husband/wife, partner, daughter, father, etc.]’ and used the person's name as appropriate. The focus was on relatives' perceptions of the experiences of the person with ABI.

### 2.4 Procedure

Ethical clearance was obtained from hospital and university ethics committees. Prospective participants were screened by a rehabilitation physician and then approached by a social worker who provided a verbal and written project summary. An interview time was scheduled with the researcher, who obtained informed consent before commencing. The female interviewer (J.M.) had a psychology background (PhD), training in interviewing people with ABI and lived experience of caring for a relative with ABI. The interviewer was unknown to participants and did not disclose her personal experiences.

T1 interviews with individuals with ABI were conducted in‐person within a private room in the inpatient rehabilitation unit. T1 interviews with family members were in‐person (69%) or via telephone (31%). All T2 interviews were conducted via telephone. Average interview duration was approximately 40 min at T1 and 24 min at T2. Four participants (P04, P06, P09, P10) had family members present during their interviews. Interviews were audiotaped and transcribed by a professional transcription service. Field notes were made after interviews.

### 2.5 Data Analysis

Analysis was informed by the five‐phase approach outlined in the framework method: familiarisation, framework development, indexing, charting and mapping, and interpretation [20, 21, 22]. NVivo (QSR International Pty Ltd, 2022) was used to facilitate analysis. The analysis focused on the self‐advocacy experiences of individuals with ABI from their own and their family members' perspectives. A hybrid deductive–inductive approach was used to develop the thematic framework whereby initial codes were informed by the study aims and interview guide but also emerged from recurring views and experiences reflected in the data.

Two authors (J.M. and T.O.) independently reviewed 25% of T1 transcripts and collaboratively developed an initial thematic framework, which was applied to the remaining T1 transcripts. These authors subsequently reviewed 25% of T2 transcripts using the initial thematic framework, which resulted in merging some codes and categories and the addition of others. The refined framework was applied to the remaining T2 transcripts. The final phases of charting and mapping and interpretation were completed by two authors (J.M. and T.O.). The framework and key findings were collaboratively reviewed by the broader research team. Quotes from people with ABI (P) and family members (F) were selected to support the findings [23].

### 2.6 Reflexivity and Rigor

Reflexivity was undertaken through preparation of a positionality statement (J.M.), use of a coding log and analysis audit trail and collaborative discussion during theme interpretation [17, 24]. Triangulation occurred through the use of multiple data sources (across time points), methods (transcripts and field notes) and investigators (academic and clinician researchers, including two with lived experience of caring for relatives with ABI).

## 3 Results

### 3.1 Sample Characteristics

Individuals with ABI (75% male) were aged 20–63 (*M* = 42.08, SD = 15.51) and had sustained their injury on average 96.25 days (SD = 57.97) before TBI or stroke (Table 2). Discharge functional independence measure scores ranged from 58 to 120 (*M* = 102.91, SD = 22.81). Family members were mainly spouses/partners (42%) or parents (33%). Two participants with ABI and three family members did not participate in T2 interviews due to health reasons or time constraints. At least one member of each dyad completed T2 interviews.

**Table 2:** Participant demographic and injury characteristics for people with ABI (P) and family member (F).

Participant dyad IDs	Age band, sex	Cause of ABI	Time since injury (days, range)	Caregiver relationship	Residential location^a^
P001 F001	50–55, male 60–65, female	TBI	151‐200	Next‐of‐kin (extended family)	Metropolitan
P002^b^ F002	40–45, male 40‐45, female	Stroke	201–250	Spouse	Rural
P003 F003^a^ F003^b^	20–25, male 45–50, male 50–55, female	TBI	0–50	Parents	Rural
P004 F004	60–65, male 65–70, female	TBI	51–100	Spouse	Metropolitan
P005 F005^b^	45–50, male 30–35, male	Stroke	101–150	Partner	Metropolitan
P006^b^ F006	60–65, male 60–65, female	Stroke	151–200	Spouse	Metropolitan
P007 F007	55–60, male 15–20, male	Stroke	0–50	Adult child	Rural
P008 F008^b^	20–25, male 35–40, female	TBI	51–100	Parent	Rural
P009 F009	45–50, female 45–50, male	Stroke	101–150	Partner	Rural
P010 F010	35–40, female 65–70, female	Stroke	0–50	Parent	Rural
P011 F011^b^	20–25, male 30–35, male	TBI	51–100	Next‐of‐kin (uncle)	Metropolitan
P012 F012	25–30, female 55–60, male	Stroke	51–100	Parent	Metropolitan

^a^ Residential locations were classified according to postcodes.

^b^ Not available or withdrew at T2.

### 3.2 Themes and Subthemes

The analysis identified an overarching theme of *reclaiming agency* and interrelated themes of *capacity meets desire* and *scaffolded support*. An overview of the themes and subthemes with supporting quotes is presented in Table 3. Self‐advocacy early after ABI reflects a process of *reclaiming agency* or people's efforts to be involved in decisions about their care and rehabilitation. Having a say in one's care is integral to human rights and dignity: ‘It's important because it makes you feel human, and it makes you feel that you matter. When you come in here, everything gets taken away from you’ (P5, T1).

**Table 3:** Overview of themes, subthemes and categories.

Themes and subthemes	Description of categories that informed themes	Example quotes (per subtheme)
*Reclaiming agency*		
Loss of agency	Lack of comprehension of injury, surroundings or care needs	‘I was pretty, still out of it, so I was just like, not really like “what's going on”, I don't know how to explain it.’ (P3, T1) ‘I was on pureed food. And I couldn't identify the food and that, and there was just small portions. So, like it kind of strengthened this thing I had in my head, that the nurses had it in for me.’ (P1, T1)
Emerging agency	Focus on current care needs; others know best	‘Look, I haven't asked any questions about going back to (hometown), because my main concentration has been to jump through the hoops that I have to jump through while I'm here.’ (P7, T1) ‘I just stayed in hospital because I knew I wasn't quite ready to leave just yet … I know I had a say, but I just went to all the different programs anyways.’ (P3, T2)
Striving for agency	Feeling empowered; planning current and future care	‘I can choose whatever time (work schedule) … It's like I know what I can do and can't do …’ (P3, T2) ‘I've started questioning who's running the finances from my fund because it'll all run out one day soon.’ (P1, T2)
*Capacity meets desire*		
Limited capacity and desire	Injury‐related changes	‘There's this, what do you call it, speech therapy. Should be given to people who can't talk here … shouldn't take people who already talk and everyone understand, you know? … I don't need speech therapy.’ (P2, T1)
Growing capacity or desire	Acceptance and adjustment; environmental impact; knowledge and experience of healthcare; postinjury rehabilitation	‘I'm only doing one step at a time, kind of thing … just take things slow … even though you're out of hospital, still rest up as much as possible.’ (P3, T2) ‘Just the one part I don't really cooperate with is I can't actually come outside by myself unless I have a visitor.’ (P8, T1) ‘The nurses know if I hear wrong information, I'm on them; “Excuse me, I didn't refuse that tablet, I wasn't offered, or I wasn't given an option”.’ (P5, T1) ‘Wasn't 'til they actually started doing work with me that I realised I needed it. Like I couldn't walk straight, and I would slur my words, and my memory was really bad.’ (P1, T1)
Realised capacity and desire	Enduring personal traits (e.g., confidence and assertiveness); focus on personal goals	‘… (after a meeting) we'll have to say at the end, “(P3), did you want to say something?” … I don't think it's to do with his complex reasoning. I think it's to do with confidence.’ (F3, T2)^a^ ‘It needs to be a mediation thing, not necessarily an arbitrary decision on my behalf where you are going to do this and I'm not going to stand for anything less.’ (P7, T2) ‘Well, I got to remember to write stuff down. Like I have appointments nearly every day … just giving me a timetable … I started putting things in order.’ (P1, T2)
*Scaffolded support*		
More directive support	Care coordination and advocacy; feeling well‐informed and prepared; emotional support	‘We were fortunate that (P3) got accepted into (transitional rehabilitation), so we'd already met with (key worker)… . it's like that continued care and what to expect.’ (F3, T2)^a^ ‘Yeah, and (insurance case manager) take over … come face‐to‐face, talk with him, assess what he needs in the present to get back to the community … I am very happy with how that was organised as support.’ (F4, T1) ‘I'm trying to get him to not feel that his life's ruined and just to give up, and the mental health… . It's going to hurt today; it's going to hurt tomorrow.’ (F11, T1)
Responsive support	Feeling listened to and heard; information and education; opportunity to ask questions; personalised, specialised care	‘(P5) asked them for that change, and they did it straight away.’ (F5, T1)^a^ ‘You get a lot information from (education sessions) … that was one thing, definitely.’ (F3, T2) ‘Being able to talk face‐to‐face is always the best thing. Spending time with you, to arrange the different things.’ (P4, T2) ‘Because most of them have experienced people with brain injuries … so they know how to work with them.’ (P3, T2)
Supported self‐management	Collaborative partnerships; ongoing support and advocacy	‘So, you definitely need a team of people, them all working together … to have your own call or at least give them options on your call. So, here's your options, these ones are flexible.’ (F11, T2) ‘There needs to be a feedback loop because if I'm not getting benefit out of it, then I need to let someone know … there's been some (support workers) that started turning up every morning for four hours, but we've since branched that back, because there's no real requirement for them to be here for that long period of time.’ (P7, T2)

^a^ Quotes reflect family members reporting on their relative's experiences or responses (person with ABI's name replaced with P5, P3, etc.).

**Figure 1:**
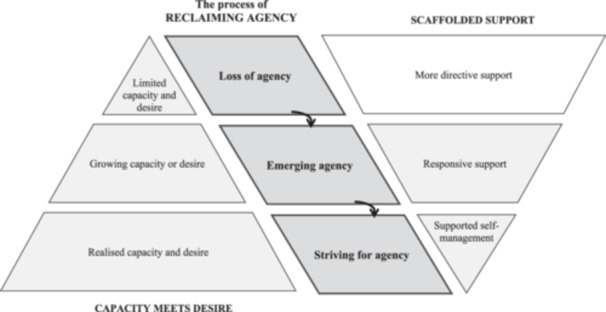
Representation of findings reflecting the process of reclaiming agency, which is typically associated with an increase in capacity and desire and a decrease in the intensity and directiveness of support.

Agency varies according to individuals' *capacity and desire* to be involved in decisions and the availability of *scaffolded support* personalised to individuals' circumstances, needs and preferences. In terms of barriers to self‐advocacy, individuals experiencing a *loss of agency* typically have *limited capacity and desire* to influence their care due to neurocognitive deficits and require *more directive support* to be kept informed about changes in their care. *Emerging agency* is facilitated by individuals' *growing capacity or desire* to influence care decisions and *responsive support*. *Striving for agency* is aided by *realised capacity and desire* to influence care decisions and *supported self‐management* to encourage individuals to direct their current and future care needs. Based on most participants' perspectives, capacity and desire gradually increase while the intensity and directiveness of support decreases (Figure 1). However, this may vary according to personal circumstances and the care context.

### 3.3 Loss of Agency

During acute recovery, individuals with ABI typically experience a loss of agency associated with impaired conscious processing of self and the environment. This affects individuals' basic knowledge and comprehension of their brain injury and care needs: ‘… even though people were telling me what's happened it's still like … what's going on? where am I?’ (P3, T1). Loss of agency is contributed to by *limited capacity and desire* to self‐advocate and necessitates *more directive support* to keep individuals informed of their care.

#### 3.3.1 Limited Capacity and Desire

Cognitive deficits such as impaired self‐awareness, memory and language initially compromise individuals' ability and motivation to question and express their care needs. A void in understanding due to loss of or fluctuating arousal persists until more consistent memories are formed: ‘I don't remember being in the main hospital much at all, I remember just being in this one here at the moment’ (P4, T1). Due to lack of comprehension of their circumstances, individuals may passively accept care decisions: ‘… he would consent to things that he would not remember consenting to’ (F7, T1). For others, a lack of comprehension of injury‐related deficits may lead to questioning or resisting care.

#### 3.3.2 More Directive Support

When agency is lost during the acute care phase, individuals with ABI rely heavily on professionals and family to communicate and coordinate their care. Family members are initially immersed in seeking information about their relative's health and want to be kept well‐informed of care transitions. They are often tasked with repeatedly orienting the person to their environment and explaining care needs, while providing essential emotional support: ‘… after he come back from ICU … (he said) “I don't know what happened, why am I in a nappy?”; he got panicky, very agitated. I just give hope and calm him down’ (F4, T1). For individuals without family advocates, care transitions may be confusing and distressing because they are less informed of or may forget about changes: ‘I'd come back to my room, but all my stuff is gone … . I had to ask the wardie—I was starting to get like anxiety … just trying to find a familiar face’ (P1, T1).

To remain well‐informed, family members are often required to proactively seek information: ‘I had to do a lot of searching and update myself because a lot of the work is handled by different people, and those people aren't working 17‐hour shifts’ (F7, T1). Family members feel better informed and able to prepare their relative for care transitions when meetings are held to explain what will happen and introduce new providers. Conversely, delayed notice or conflicting information affects family members' ability to communicate with and prepare the person with ABI for change: ‘The nurses told me they already approved that transfer … and then the doctor upstairs there said “Oh, we don't know when he is going to move”’ (F5, T1).

As close family know the person with ABI best, they can communicate their relative's care preferences when they are unable to do so themselves: ‘I have to explain to them why, when they said to me, “he's refused this” … where they were going wrong, and so it's not the best approach’ (F11, T1). Insufficient explanation for care transitions may contribute to individuals feeling they have little choice over care decisions: ‘Maybe instead of saying, “We're going to put you in this next ward” They could say: “The next stage, if you think it would be good for you or if that is what you like, then we can move you into (rehabilitation ward)”’ (P12, T1).

Hospital discharge represents a major adjustment due to shifts in responsibility for care coordination. Although professionals aim to prepare individuals and their families for this transition, they may still feel unprepared for the loss of structured support: ‘You get well looked after in hospital, then coming home to looking after yourself is always a challenge’ (P4, T2).

### 3.4 Emerging Agency

Agency begins to emerge when individuals with ABI grasp the need for care and rehabilitation. Initially, this might reflect a general understanding that rehabilitation will assist them, rather than specific understanding of their care needs: ‘Just makes you feel more of in a safe space … that you're actually going to get something out of rehab and being here’ (P12, T1). As a result, individuals are initially focused on current care decisions affecting them in the here‐and‐now and may not feel ready to consider future care needs. Individuals may recognise professionals' knowledge and defer to their judgement of what is needed: ‘I suppose you've got to do as the doctor tells you’ (P6, T1). They may also rely on family members to advocate for their needs: ‘I just can't be bothered (laughter) … I feel he's got more like an understanding … it's just easier’ (P12, T1).

#### 3.4.1 Growing Capacity or Desire

Capacity and desire to self‐advocate increases with cognitive recovery and growing recognition of postinjury deficits. Accepting and adjusting to ABI means learning to be patient in the recovery process and recognising new limits. Increased self‐awareness may be signalled by individuals adjusting their self‐expectations and recognising the need for rehabilitation: ‘… where you could swim 50 metres, well, you can't swim 50 metres anymore, be lucky to do 20 metres’ (P4, T2). Participation in rehabilitation can also facilitate self‐awareness, reinforcing the benefits of participation. Recognising the need for compensatory strategies is a concrete indicator of self‐awareness: ‘Just trying to write notes and trying my best to absorb the conversations’ (P10, T1). Mental health concerns can arise when individuals perceive they are not progressing at the rate they expected: ‘It did affect him quite badly, I think from a mental health point of view … especially now getting to the five‐month mark’ (F6, T1). In turn, mental health issues can influence individuals' motivation to self‐advocate.

The hospital environment also affects individuals' capacity and desire to self‐advocate because it can feel overly restrictive, particularly during extended admissions: ‘It reminded me of jail a lot at first … just that format you've got to get into, like dinners are all at the same time. I wasn't allowed out’ (P1, T1). The structured support in hospital can also mask postinjury impairments, whereas home visits and discharge facilitate understanding of deficits: ‘Because the hospital is like a big routine … then you get to explore the open world again … . You get mentally fatigued really easily’ (P3, T2).

For some, their ABI represents an abrupt introduction to medical care and healthcare systems. Individuals' limited knowledge or experiences can adversely affect their understanding of their rights and confidence in decision‐making: ‘I'd never been to a doctor and all of a sudden they were sticking in needles and tubes’ (P5, T1). Conversely, those with existing knowledge and experience of healthcare are better positioned to know what to expect and how to advocate for care changes: ‘I'm a nurse … so I talked to the doctor and they listened to me … and instead used a lower dose of drug for him’ (F5, T1).

#### 3.4.2 Responsive Support

Emerging agency is facilitated by responsive support that addresses individual's current care needs. This involves feeling listened to and heard by professionals, having the opportunity to ask questions and perceiving support is readily available. Timely access to information and education about ABI is central to increasing individuals' understanding of their injury as well as their families'. This extends to information regarding what to expect from different services: ‘The physio … she went through it, no one else did. I've got a better understanding now’ (P5, T2). Some individuals benefit from peer support or advice from others with ABI: ‘… someone you know that has a brain injury … I ask questions … I had a few recommendations I could choose’ (P3, T2).

Positive perceptions of care relate to being treated with dignity and respect by professionals and receiving support personalised to one's background and family needs: ‘The Aboriginal liaison office here is to be just so highly commended … they took us in, they looked after us, they made sure that I got accommodation’ (F10, T1). Responsive support is enhanced by relationship building between professionals and individuals with ABI: ‘Every day you see them, talk to them, it becomes like you know them, you know?’ (P2, T1). Having professionals experienced with brain injury instill greater confidence in care. Conversely, negative experiences of care occur when individuals with ABI do not feel heard or understood by professionals: ‘And some people just smile and mutter shit under their breath. Even when I try to explain it, like I need help… “Yeah we know”—I was like, you don't know the pain I'm in’ (P11, T1).

### 3.5 Striving for Agency

Agency is most apparent when individuals with ABI begin to plan and direct their future care. Although more evident postdischarge, some individuals realise their capacity to influence their future care while in‐hospital, supported by structured processes (e.g., care coordination meetings). Yet, agency may be reduced in new situations following discharge, due to unfamiliar processes and complex support systems. Therefore, self‐advocacy is a relative phenomenon that varies according to the care context and reflects individuals' ongoing efforts to reclaim agency after ABI.

Striving for agency involves feeling empowered to influence and direct one's current and future care and perceiving that one has choice and control: ‘… so I can say no I can't make it if I've got something on. Or I'll take my time or I'm doing really well with physio’ (P5, T2). Feeling more in control of one's life means solving issues that arise, and planning and self‐advocating for one's future needs: ‘I'll get myself organised and I'll get whatever pension that I'm entitled to organised, and that'll keep food in my belly’ (P7, T1).

#### 3.5.1 Realised Capacity and Desire

As individuals with ABI become increasingly aware of postinjury changes and understand their ongoing needs, they are more motivated to direct their own care. This is particularly the case for support relevant to achieving personal goals, such as independence: ‘So, I do have someone lined up when [partner's] back at work that can come out and help me and drive me around … it's something that I probably will need.’ (P9, T2). Individuals' personal characteristics, like self‐confidence and assertiveness, influence their desire to self‐advocate: ‘I'm getting a bit more control back … I just had to take the reins; you know?’ (P5, T2).

#### 3.5.2 Supported Self‐Management

As individuals with ABI become more focused on their future care needs, decision‐making is more collaborative, and support is faded to foster self‐management. Individuals may continue to seek advice from professionals and draw upon their informal support networks. However, these relationships reflect more of a partnership approach in which individuals are given choices and encouraged to make decisions: ‘Last time, she asked if I needed—wanted to go to a speech session. I said to her that I don't think that I need to, but still went, just to see if I am improving in it or not’ (P8, T2).

Self‐management is facilitated through ongoing support and stability in care providers who understand the person's functional capacity and seek to empower them with greater responsibility for decision‐making. Obstacles to accessing support can be a catalyst for change, propelling individuals to assert themselves and question others' decisions: ‘I had to say I want to decide who I have (therapists) … we are choosing physio, which is very important’ (P5, T2). Over time, individuals with ABI may take greater responsibility for communicating and influencing their support needs.

Although family advocacy is especially prominent during hospital‐based care, it continues to varying degrees post‐discharge. Some families may feel able to step back from their advocacy role: ‘(my partner) said if you don't want to do it, don't do it. I'm not going to force you, you know what you're doing’ (P5, T2). However, the balance between self‐ and family advocacy can be challenging due to changes in relationship dynamics, associated, for example, with managing carer responsibilities within romantic relationships or parents wanting to protect yet empower their adult child: ‘So, he's starting to take more and more ownership … but sometimes we just have to step in and go—like, this isn't working … we kind of linger in the background’ (F3, T2). At times, individuals with ABI may be more receptive to advice from trusted professionals than their family members.

Ultimately, self‐advocacy is considered a vital skill to ensure individuals can access the support needed to optimise their recovery and quality of life: ‘It's what I need to be able to function outside of here … I need to know what my care and rehabilitation has been here to be able to continue when I get home. It's everything’ (P10, T1).

## 4 Discussion

The current findings highlight that, early after ABI, self‐advocacy reflects an ongoing process of reclaiming agency over decisions about one's care. Level of agency varies according to individuals' capacity and desire to be involved in these decisions and the availability of scaffolded support. Key barriers to self‐advocacy for individuals with ABI include neurocognitive deficits, unfamiliar and highly structured environments and lack of family support. Conversely, self‐advocacy is facilitated by neurocognitive recovery, increased self‐awareness of postinjury changes, a growing desire to understand and influence one's care and availability of scaffolded support from family and clinicians. The development of self‐advocacy or ‘striving for agency’ is most apparent postdischarge, as individuals typically feel more empowered to influence and direct their current and future care, although this varies according to the care context and individuals' characteristics.

The current conceptualisation of reclaiming agency over care decisions builds upon previous research exploring individuals' early understanding of their ABI in hospital and their care in the community [7, 13, 25, 26]. Carrier, Ponsford and McKay [26] identified families' integral role in advocating for early care needs of individuals with ABI, due to initial neurocognitive changes. Gabbe et al. [25] similarly found that communication and memory difficulties limited individuals' engagement in decision‐making during their acute care. Care coordination, timely access to services and clinician consistency helped people to stay informed of their postdischarge care. Focusing on individuals with ABI living in the community, a systematic review by Murray et al. [13] identified that readiness to reclaim choice and control was a gradual process facilitated by and negotiated with health professionals and supporters [13]. Extending on these accounts, the current research identified person‐level and contextual barriers and facilitators to individuals developing self‐advocacy skills during hospital and early postdischarge care. Specifically, neurocognitive deficits and highly structured and unfamiliar environments reduce individuals' capacity and desire to advocate for their care needs, which is further compromised by a lack of family support. Conversely, the ability to influence and direct one's current and future care is facilitated by neurocognitive recovery, a growing desire to self‐advocate and scaffolded support from family and clinicians.

The current findings also build on person‐centred frameworks [5, 14, 27], which highlight existing clinical approaches for increasing people's participation in care decisions such as collaborative goal setting and supported decision‐making. Clinically, they highlight the scope to embed self‐advocacy skills early in brain injury rehabilitation. Guided by the central theme of reclaiming agency (see Figure 1), there is a need for tailored strategies to address the barriers to self‐advocacy that we found were associated with each level of agency. Informed by the current findings (i.e., perceptions of what helped and the suggestions of participants with ABI and family members), evidence from the literature and the authors' own expertise, Table 4 provides an overview of barriers to reclaiming agency and associated strategies for enhancing self‐advocacy skills throughout hospital transitions and into the community. Overarching principles relate to providing care that is personalised to individuals' background and family needs, promoting individuals' dignity and ability to enact their rights, and building relationships and collaborative partnerships in care. Given that the absence of family support was a key barrier to reclaiming agency, individuals with ABI without family support may benefit from greater clinician support or a communication liaison officer to stay informed of and participate in care decisions.

**Table 4:** Barriers to reclaiming agency and strategies for enhancing self‐advocacy skills according to level of agency.

Agency	Barriers to agency	Strategies for supporting self‐advocacy
Loss of agency	Neurocognitive impairment (e.g., low arousal, agitation and inability to retain new information) Highly structured, unfamiliar and stressful environment Staffing changes (e.g., shifts and rotations) and gaps in information and care continuity Absence of family support or advocacy	More directive support: Reinforcing key information about the injury and care context, supported by reminders in the environment (e.g., visual aids) Reminders and explanations of care procedure just before their delivery (verbal and visual communication aids for upcoming procedures) Clear communication between professionals regarding an individual's care needs to ensure consistency with communication and continuity of care for the person with ABI and their family Building relationships by seeking information regarding the person's personality, preferences and optimal communication approaches from family and previous treating professionals Increasing sense of safety during and after care transitions through family engagement and emotional support Monitoring the person's readiness for new information, offering choices regarding timing and format (e.g., verbal and written) and encouraging questions Regular information exchange with family members (who typically act as an information channel for the person with ABI) Linking individuals to communication liaison officers, particularly in the absence of family support
Emerging agency	Persisting neurocognitive deficits and impaired self‐awareness Difficulty accepting and adjusting to the ABI and unrealistic expectations for recovery Limited knowledge or experience with healthcare system Loss of autonomy in day‐to‐day activities due to restrictiveness of hospital environment	Responsive support: Exploring what is most important to the individual with ABI and family in the here‐and‐now, to ensure personalised care and facilitate collaborative goal‐setting or planning relevant to personally relevant outcomes Having a clear point of contact, providing regular opportunities for questions and responding promptly to requests for information and clarification Holding care transition meetings to explain what will happen and introduce new care providers (e.g., preparing for a change in structure of support post‐discharge) Ensuring timely access to education about brain injury in preferred formats, with spacing of time to allow people to process information and ask questions Facilitating opportunities for peer support from peers who are at a similar or future stage in their journeys Outlining the role of services, patients' rights and scope for choice over care providers and access to rehabilitation Graduated return to and exposure to ‘the real world’ to learn about postinjury changes and test one's limits (e.g., home visits) Reinforcing developments in self‐awareness (e.g., altered self‐expectations, increased engagement in rehabilitation, strategy use) and supporting emotional reactions to growing insight into post‐injury changes Monitoring individual's readiness to engage in discussions and make decisions regarding future care needs
Striving for agency	Persisting neurocognitive deficits and adjustment‐related difficulties Lack of self‐confidence and assertiveness Overreliance on advocacy from family or professionals Self‐ vs. family‐advocacy: tension between desire to empower vs. protect	Supported self‐management: Initially, providing more structured opportunities for individuals to identify their care needs and be involved in future care decisions (e.g., discharge support planning) Linking individuals' personal goals to current and future support needs to increase personal relevance and motivation to engage in care decisions Fading support to recognise individuals' growing capacity to identify and solve problems, choose different support options and communicate their decisions Encouraging individuals to view challenges to accessing support as an opportunity for them to take more responsibility by exercising choice and control over their care Educating and supporting family members' understanding of 'he benefits and limits of supported self‐management

As outlined in Table 4, more directive support is recommended for individuals experiencing a loss of agency during inpatient rehabilitation, whereas responsive support may be optimal to support preparation for care transitions including discharge. However, judgement is needed regarding the timing and suitability of strategies for each individual. Memory retention is limited until posttraumatic amnesia (PTA) resolves, supported by maintaining a quiet, safe and consistent environment [28]. More directive support strategies such as orientation cues, explanations of care procedure just before their delivery and emotional support are most appropriate during PTA [26, 28]. Clear communication, collaborative decision‐making and having a positive attitude towards enabling individuals with ABI to self‐advocate are key across levels of agency and rehabilitation settings [5, 13, 29]. Stanley et al. [29] found that professionals can experience tension in supporting dignity of participation whilst managing risk. When individuals' choices and decisions are considered unsafe or against their best interests, professionals can support dignity of risk by providing learning opportunities for failure and success within rehabilitation. Mäkelä, Gawned and Jones [15] similarly identified that staff attitudes and beliefs regarding self‐management (e.g., patient ownership of rehabilitation goals) influenced whether they implemented a stroke self‐management package in acute services.

Opportunities to learn, practice and apply self‐advocacy skills need to be coordinated and integrated across the rehabilitation journey, as individuals face novel challenges within the complex health and disability services interface [2, 4]. Peer‐based self‐advocacy skill training programmes have been found to increase self‐efficacy and life satisfaction in long‐term adjustment [30, 31]. Further research is needed to develop and evaluate a systematic approach for embedding self‐advocacy skill development early in brain injury rehabilitation. Although not the focus of the current study, family members' own challenges in accessing timely information, being involved in care decisions and balancing desire to empower yet protect their relative emerged in the findings. As identified in previous research [32, 33], family members' support needs during hospitalisation and community reintegration are essential to address given their considerable stress, which impacts their own well‐being and ability to be effective advocates.

### 4.1 Limitations and Future Research Directions

While the current findings advance understanding of early experiences of self‐advocacy after ABI, some limitations may influence their relevance and transferability to other health service contexts, particularly given the single recruitment site. Despite the inclusion of participants with diverse sociodemographic and injury characteristics, the perspectives of individuals with severe aphasia and low English fluency from culturally and linguistically diverse backgrounds were not represented. Similarly, all participants with ABI had a participating family member; hence, their perspectives may not adequately reflect those without family support. Further, while perspectives of both individuals with ABI and family members were gained to enhance the credibility of the findings, member checking was not conducted. In future, it would be valuable to explore clinicians' and service managers' perspectives regarding barriers and facilitators to self‐advocacy skill development early in rehabilitation. Family members' experiences of barriers to advocating for their own needs in conjunction with supporting the individual with ABI is also an important future focus of research. Importantly, this study was conducted in a specific rehabilitation setting, which may affect the transferability of findings. Barriers to self‐advocacy are likely to vary according to sociocultural and policy factors that affect care transitions and pathways. It would be beneficial to explore barriers and facilitators to self‐advocacy across different healthcare systems and cultures. Further, examining self‐advocacy experiences during other care transitions (e.g., between ABI‐specific and general disability services) over a longer timeframe would be useful.

An early focus on the development of self‐advocacy skills in the rehabilitation journey is likely to promote dignified care that meets individuals' personal needs and preferences and can be fostered across contexts relevant to achieving their goals. At a systems level, individuals' greater participation in care decisions has the potential to improve the efficiency of clinical handovers and avoid discharge delays [4].

## 5 Conclusion

Based on the perspectives of individuals with ABI and their family members, this study highlighted that self‐advocacy after ABI entails a process of reclaiming agency whereby individuals seek to understand, question and direct their ongoing care. Barriers to self‐advocacy early after ABI include neurocognitive deficits, unfamiliar and highly structured environments and lack of family support. A framework of strategies was presented to support clinicians, family members and other advocates to initiate the process of building agency early in individuals' recovery. Research incorporating clinician perspectives and feedback on the proposed framework is recommended.

## Author Contributions

**Tamara Ownsworth:** conceptualisation, investigation, funding acquisition, writing–original draft, methodology, validation, visualisation, writing–review and editing, supervision, resources, formal analysis. **Annerley Bates:** conceptualisation, investigation, funding acquisition, writing–review and editing, methodology, supervision, resources, validation. **Kerrin Watter:** conceptualisation, investigation, funding acquisition, writing–review and editing, methodology, resources, validation. **Clare Morgan:** conceptualisation, investigation, funding acquisition, methodology, writing–review and editing, resources, validation. **Ryan Bell:** conceptualisation, investigation, funding acquisition, writing–review and editing, resources, methodology. **Janelle Griffin:** conceptualisation, investigation, funding acquisition, writing–review and editing, methodology, resources. **Ben Turner:** conceptualisation, investigation, funding acquisition, writing–review and editing, methodology, resources, validation. **Areti Kennedy:** conceptualisation, investigation, funding acquisition, writing–review and editing, methodology. **Melissa Kendall:** conceptualisation, investigation, funding acquisition, methodology, writing–review and editing, validation. **Belinda Adams:** conceptualisation, methodology, validation, writing–review and editing, investigation, funding acquisition. **Emily Gibson:** conceptualisation, investigation, funding acquisition, writing–review and editing. **Troy Hakala:** conceptualisation, investigation, funding acquisition, methodology, validation, resources, writing–review and editing. **Jessie Mitchell:** investigation, writing–original draft, methodology, validation, visualisation, writing–review and editing, software, formal analysis, project administration, data curation, resources, conceptualisation.

## Ethics Statement

This study was performed in line with the principles of the Declaration of Helsinki. Approval was granted by the Ethics Committees of Metro South Health (HREC/2022/QMS/83676) and Griffith University (GU Ref. No.: 2022/265).

## Consent

Informed consent was obtained from all individual participants included in the study.

## Conflicts of Interest

The authors declare no conflicts of interest.

## Data Availability

The data that support the findings of this study are available from the corresponding author upon reasonable request.
